# Niveles de ruido generados en procedimientos realizados en una facultad de odontología

**DOI:** 10.15649/cuidarte.2251

**Published:** 2022-08-27

**Authors:** Andrea Patricia Acuña Vesga, Leidy Carine Díaz Ramírez, Andrea Johanna Almario Barrera, Adriana Esperanza Peñuela Sánchez, Yeny Zulay Castellanos Domínguez

**Affiliations:** 1 Estudiante de odontología. Universidad Santo Tomás seccional Bucaramanga, Colombia. Email: aavesga@hotmail.com Universidad Santo Tomás Universidad Santo Tomás Bucaramanga Colombia aavesga@hotmail.com; 2 Estudiante de odontología. Universidad Santo Tomás seccional Bucaramanga. Colombia. Email: leidy.diaz01@ustabuca.edu.co Universidad Santo Tomás Universidad Santo Tomás Bucaramanga Colombia leidy.diaz01@ustabuca.edu.co; 3 Odontóloga, docente investigadora grupo Salud Integral Bucal facultad de Odontología de la Universidad Santo Tomás seccional Bucaramanga, Colombia. Email: andrea.almario@ustabuca.edu.co Universidad Santo Tomás facultad de Odontología Universidad Santo Tomás Bucaramanga Colombia andrea.almario@ustabuca.edu.co; 4 Médico veterinaria, magíster en epidemiología, docente Facultad de odontología Universidad Santo Tomás seccional Bucaramanga, Colombia. Email: andrea.almario@ustabuca.edu.co Universidad Santo Tomás Facultad de odontología Universidad Santo Tomás Bucaramanga Colombia andrea.almario@ustabuca.edu.co; 5 Bacterióloga, magíster en epidemiología, docente investigadora grupo Salud Integral Bucal Facultad de odontología Universidad Santo Tomás seccional Bucaramanga Colombia. Email: yeny.castellanos@ustabuca.edu.co Universidad Santo Tomás Facultad de odontología Universidad Santo Tomás Bucaramanga Colombia yeny.castellanos@ustabuca.edu.co

**Keywords:** odontología, ruido, universidades, salud laboral, dentistry, noise, universities, occupational health, odontologia, ruído, universidades, saúde do trabalhador

## Abstract

**Introducción:**

Los profesionales del área de odontología se ven expuestos a diferentes tipos de ruidos generados en el ambiente laboral producidos durante el ejercicio de su práctica clínica, originados, entre otros, por el instrumental y los aparatos rotatorios de uso diario. Estos niveles de ruido pueden variar de acuerdo a la especialidad clínica.

**Objetivo:**

Determinar el nivel de ruido producido durante los procedimientos odontológicos en las clínicas de una facultad de odontología.

**Material y Métodos:**

A partir de un estudio de corte transversal analítico y mediante muestreo no probabilístico a conveniencia se realizó el reconocimiento de las unidades en las especialidades odontológicas a evaluar. Se usó un sonómetro digital BENETECH GM1352, nivel de frecuencia A, rango 30-130 dB, exactitud más o menos 1,5 dB. Se utilizaron las pruebas U de Mann Whitney y Kruskal Wallis para determinar diferencias en los niveles de ruido entre las especialidades odontológicas.

**Resultados:**

La mediana del nivel de ruido medido en general fue de 75,94 dB (RIC 74,12 - 77,51), la especialidad clínica en la que mayor ruido se identificó fue la operatoria dental (mediana 77,34 y RIC 76,44 -79,4 dB).

**Conclusiones:**

las áreas clínicas operatoria dental, rehabilitación, endodoncia y odontopediatría corresponden a las especialidades donde los niveles de ruido determinados se ajustan a los límites permitidos por la normatividad vigente en Colombia para el ruido medido en ambiente laboral.

## Abstract (en)

**Introduction:**

Dental professionals are exposed to different noise levels in their work environment during their clinical practice, mainly caused by dental instruments and rotary instruments used on a daily basis. Noise levels may vary according to the type of clinical specialty.

**Objective:**

To determine noise levels during dental procedures in dental school clinics.

**Materials and Methods:**

An analytical cross-sectional study was conducted by means of non- probability convenience sampling to determine dental specialties to be evaluated. BENETECH GM1352 30-130dB Digital Sound Level Meter with accuracy +/- 1.5 dB and A weighting was used for measurements. Mann-Whitney U and Kruskal-Wallis H tests were used to identify differences in noise levels among dental specialties.

**Results:**

Measured median noise level was 75.94 dB (RCI 74.21 -77.51), dental surgery was identified to have the highest noise among clinical specialties (median 77.34 and RCI 76.44 -79.4 dB).

**Conclusions:**

Dental surgery, oral rehabilitation, endodontics and pediatric dentistry were found to be the clinical specialties where noise exposure is within the limits established by Colombian regulations for noise in the workplace.

## Resumen

**Introdução:**

Os profissionais da área da odontologia estão expostos a diferentes tipos de ruído gerado no ambiente de trabalho produzido durante o exercício da sua prática clínica, proveniente, entre outros, de instrumentos e aparelhos rotativos em uso diário. Estes níveis de ruído podem variar de acordo com a especialidade clínica.**Objetivo:** Determinar o nível de ruído produzido durante procedimentos odontológicos nas clínicas de uma escola de odontologia.

**Material e Métodos:**

Com base em um estudo analítico transversal e por meio de amostragem por conveniência não probabilística, foram levantadas as unidades das especialidades odontológicas a serem avaliadas. Foi utilizado um sonómetro digital BENETECH GM1352, nível de frequência A, faixa de medição 30-130 dB, precisão mais ou menos 1,5 dB. Os testes U de Mann Whitney e Kruskal Wallis foram usados para determinar as diferenças nos níveis de ruído entre as especialidades odontológicas.

**Resultados:**

O nível de ruído médio medido globalmente foi de 75,94 dB (RIC 74,12 - 77,51), sendo que a especialidade clínica em que foi identificado o maior ruído foi a cirurgia dentária (média 77,34 e RIC 76,44 -79,4 dB).

**Conclusões:**

as áreas clínicas de cirurgia dentária, reabilitação, endodontia e odontologia pediátrica correspondem às especialidades onde os níveis de ruído determinados estão de acordo com os limites permitidos pela regulamentação em vigor na Colômbia para o ruído medido no ambiente de trabalho.

## Introducción

En la última década, se estima que más del 80% de la población ha estado expuesta a niveles de ruido superiores a 85 decibeles (dB) durante el ejercicio de su ocupación laboral. Así, la exposición al ruido se constituye en un factor de riesgo para la calidad de vida, representando cerca de seis millones de años de vida perdidos por discapacidad (AVADS) para el año 2017^1^^,^^2^. A nivel mundial, casi 400 millones de personas presentan algún grado de lesión auditiva producto de la exposición a las altas ondas sonoras generadas en el ambiente en que laboran. De acuerdo con la Organización Mundial de la Salud (OMS), la exposición a sonidos elevados en el entorno laboral es una de las causas de pérdida de la audición^3^.

La hipoacusia ocupa el tercer lugar en enfermedades que involucran AVADS^4^ y la segunda causa más común de pérdida auditiva neurosensorial^5^. La capacidad auditiva puede verse afectada por la exposición al ruido. De acuerdo con los estándares internacionales, los decibeles (dB) soportados por el oído humano sin provocar daños son 80 dB por ocho horas diarias^6^, siendo el nivel máximo permitido de exposición de 115 dB^7^. Adicionalmente, el oído necesita como mínimo 16 horas de descanso para recuperarse de 120 minutos de exposición a 100 dB^8^.

Los profesionales del área de odontología en su ejercicio clínico se encuentran expuestos de forma permanente a ruidos generados a partir de aparatos rotatorios que se utilizan en el ejercicio de la práctica clínica tales como la pieza de alta, micromotores, turbinas, entre otros, considerando además que la exposición a éstas fuentes ruidosas inicia desde el proceso de formación en las prácticas profesionales^9^. La evidencia científica demuestra que el umbral de audición de los profesionales en odontología es inferior en comparación con otro tipo de personal que apoya el trabajo en el área dental^10^. Así mismo, se ha documentado que a mayor tiempo de exposición al ruido mayor es el riego de daños auditivos irreversibles^11^^,^^12^. Se ha reportado que el nivel de ruido tiene variaciones conforme a la especialidad clínica, en donde los procedimientos de operatoria dental corresponden a los que más ruido producen, como consecuencia del uso repetitivo de fresas además de la realización de cortes en las estructuras dentales, generando niveles de ruido que pueden superar los 80 dB^13^. Por lo anterior, esta población es susceptible a desarrollar patologías auditivas derivadas de ésta exposición tales como hipoacusia y tinnitus otros^14^.

Aunque la normatividad nacional vigente es clara respecto a los niveles de exposición máximos permitidos (de hasta 115 dB)^7^, se desconocen los niveles de ruido que se generan en la práctica profesional y por lo tanto a los que se exponen tanto estudiantes como docentes y auxiliares de una facultad de odontología. Así, el objetivo de este estudio fue determinar el nivel de ruido producido durante los procedimientos clínicos odontológicos en las clínicas de una facultad de odontología en Colombia en las especialidades de operatoria dental, rehabilitación oral, odontopediatría y endodoncia.

## Materiales y Métodos

Se realizó un estudio analítico de corte transversal a partir de las mediciones de niveles de ruido detectados en las clínicas odontológicas de la facultad de odontología de la Universidad Santo Tomás sede Floridablanca donde se atienden pacientes para diferentes especialidades clínicas. Se compararon los niveles de ruido generados durante los procedimientos odontológicos conforme al área clínica en la que se encuentran expuestos tanto docentes como estudiantes durante la consulta^15^. Las mediciones se realizaron entre agosto y octubre del año 2019, antes de la reducción en el flujo de pacientes debido a la pandemia por COVID-19, en las diferentes áreas de consulta para odontopediatría, rehabilitación oral, endodoncia y operatoria dental. Estas especialidades se realizan entre el segundo y cuarto piso de las clínicas las cuales disponen de 45 unidades habilitadas en cada piso, para un total de 135 unidades.

Mediante muestreo no probabilístico a conveniencia fueron seleccionadas las unidades odontológicas donde se realizaron las mediciones del ruido. Las mismas se realizaron en aquellas unidades en funcionamiento. Para cada área clínica se realizaron tres mediciones en tres momentos diferentes por semana hasta cubrir lo correspondiente a cuatro semanas los días jueves viernes y sábado. Los puntos donde se recogieron y determinaron los niveles de ruido fueron junto a cada unidad; el sonómetro fue ubicado 1,20 metros del piso, 1,50 metros de la parte externa del cabezal de la unidad y 0,5 entre el investigador y el sonómetro, garantizando que el micrófono estuviera orientado en la dirección de la fuente sonora específica. Las mediciones se realizaron con intervalos de un minuto hasta completar 5 minutos; con lo anterior se pudo estimar el promedio de la medición de ruido.

Para el registro de las mediciones se usó el sonómetro digital marca BENETECH GM1352, el cual tiene un nivel de frecuencia A, Rango 30 a 130 dB, exactitud más o menos 1,5 dB; para la toma de las mediciones de ruido ambiental, se siguieron las recomendaciones suministradas por el fabricante. Las mediciones fueron realizadas por un experto en el manejo y uso del dispositivo.

Al momento de la obtención de las mediciones se garantizó que hubiera el mínimo de personas presentes en la zona y que fueran ajenas a la práctica además que se encontraran lo más separadas del instrumento a fin de evitar el apantallamiento del micrófono^16^.

Finalmente, los datos obtenidos se digitaron por duplicado y de forma independiente en Microsoft Excel por dos investigadoras. La información validada se exportó al paquete estadístico Stata/MP versión 14.0. para el procesamiento de los datos. La base de datos fue almacenada en Mendeley Data^17^.

El análisis estadístico incluyó un análisis univariado, en el que las variables de cuantitativas se resumen con medidas de tendencia central (media o mediana) y medidas de dispersión (desviación estándar [DE] o rango intercuartílico [RIQ]), la normalidad de éstas variables se estableció con el test de Shapiro wilk. En el caso de las variables cualitativas, se usaron las frecuencias absolutas y relativas. El análisis bivariado determinó la relación entre nivel de ruido (variable dependiente) frente a las variables independientes tales como la especialidad, la jornada y el día de la semana en el que se realizaron las mediciones. Así, para la evaluación de las variables independientes y la variable dependiente se usó la prueba de Anova/Kruskal Wallis. Valores de p menores a 0,05 fueron considerados de significancia estadística.

De acuerdo con la resolución 08430 de 1993 que rige la investigación en Colombia, esta investigación respetó los principios establecidos en cuanto al cumplimiento de las normas científicas y administrativas para las investigaciones en salud^18^. Adicionalmente, este estudio se sometió a revisión por parte del comité de ética de la Facultad de Odontología de Universidad Santo Tomás seccional Bucaramanga fue avalado mediante acta número 114022019.

## Resultados

Se realizaron en total 48 mediciones del ruido generado en las especialidades de las áreas clínicas odontológicas; la mayoría de éstas se registraron en la jornada de la mañana (68,75%) y el día viernes (50%) (Tabla 1). El comportamiento de las mediciones de ruido no tuvo una distribución normal.

**Tabla 1:** Descripción de las medidas de ruido obtenidas de acuerdo al área clínica

Variable	N mediciones repetidas (%)	Mediana	RIC	Valor de p
Especialidad clínica				<0,001
Operatoria dental	12 (25)	77,34	76,44-79-40	
Odontopediatría	12 (25)	77,0	75,05-82,44	
Rehabilitación	12 (25)	75,65	73,66-76,68	
Endodoncia	12 (25)	73,82	71,88-74,35	
Día				<0,001
Jueves	12 (25)	77,34	76,44-79,40	
Viernes	24 (50)	76,30	74,57 - 77,51	
Sábado	12 (25)	73,82	71,88 - 74,35	

**Figura 1:**
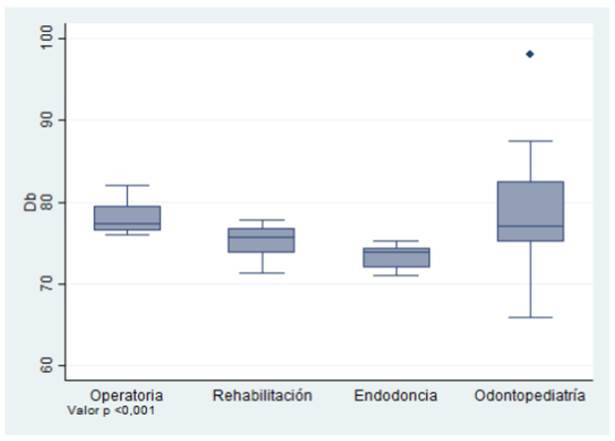
Ruido medido en decibeles, registrado de acuerdo a la especialidad clínica

De las mediciones realizadas, el nivel de ruido mínimo detectado correspondió en promedio a 71,9 dB (valores mínimo y máximo: 69,8-74,25 dB) y la máxima fue de 80,0 dB (valores mínimo y máximo: 77-82,2 dB). En general, la mediana del ruido para todas las áreas clínicas fue de 75,94 dB (RIC 74,12 - 77,51 dB), siendo la clínica de operatoria dental la que más ruido registró durante la consulta odontológica (valor de p< 0,001 determinado con test de Kruskal Wallis) (Tabla 1, Figura 1).

En cuanto al análisis de ruido de acuerdo a la jornada, no se evidenciaron diferencias entre la mañana y la tarde (p= 0,193), tampoco hubo diferencias estadísticas en las mediciones reportadas de acuerdo con el piso de la clínica en el que son desempeñadas las actividades clínicas (p= 0,089) (Tabla 2).

**Tabla 2:** Ruido registrado en las clínicas odontológicas de acuerdo a la jornada y piso de la clínica.

	N° mediciones repetidas (%)	Mediana (dB)	RIC	Valor p
Jornada				0,193
Mañana	33 (68,75)	76,3	74,24- 78,48	
Tarde	15 (31,25)	75,24	73,56- 76-38	
Piso de la clínica				0,089
Segundo	12 (25)	75,65	73,66- 76,68	
Tercero	12 (25)	74,35	73,32- 76,76	
Cuarto	12 (25)	76,83	75,98- 79,12	

**Figura 2:**
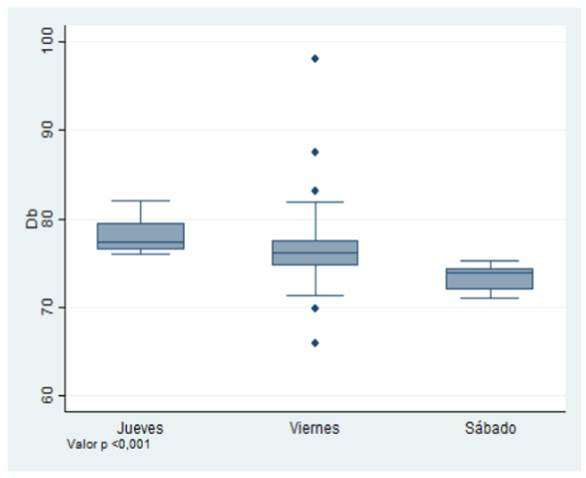
Ruido medido en decibeles, registrado de acuerdo al día de la semana en que se realizó la medición.

Por su parte, el día jueves registró niveles de ruido de 77,34 dB siendo este el día con mayor exposición al ruido con relación a los otros dos (p< 0,001 determinado con test de Kruskal Wallis) (Figura 2).

## Discusión

Esta investigación determinó los niveles de ruido que se detectan a partir de mediciones ambientales de acuerdo con la especialidad clínica donde se tuvo en cuenta además la jornada, día y piso en el que se llevó acabo el procedimiento odontológico.

Teniendo en cuenta que el personal docente así como los estudiantes se encuentran en el ejercicio asistencial un promedio de cuatro horas al día y que los registros sonoros en este estudio determinados durante la atención odontológica estuvieron por debajo de los 95 dB, se puede confirmar que los niveles de ruido están dentro de los límites aceptados por la normatividad nacional colombiana^7^ lo que a su vez es consistente con los hallazgos reportados en otras investigaciones de este tipo^19^.

El estudio publicado por Lozano y colaboradores presenta los resultados de mediciones de ruido en la facultad de odontología de una universidad peruana; los autores encontraron niveles de ruido que oscilaron entre 61,62 dB y 83,13 dB; cabe señalar que las mediciones se realizaron tres veces seguidas por tres segundos y a una distancia del operador de 45 cm ubicando el sonómetro a nivel del oído de éste^13^; las mediciones registradas en este trabajo estuvieron entre 71,9 dB y 80 dB lo cual es semejante a los registros sonoros de Lozano aún cuando las condiciones de medición variaron en lo que respecta a la ubicación del sonómetro, la unidad y el operador así como las frecuencia en la toma de las mediciones.

Un estudio realizado por Cunha y Santos midió la intensidad de ruido durante 5 minutos en el tiempo basal, antes de empezar la clínica y luego a segunda y tercera hora de actividad clínica, se tomaron 10 mediciones repetidas en un intervalo de 1 semana, el decibelímetro que se utilizó se encendió por 5 minutos y la medida que tuvo mayor intensidad fue la que se registraba, solo un examinador apuntó los resultados sin mirar registros anteriores^12^.

La especialidad con el mayor nivel de ruido producido fue operatoria dental, lo que también coincidió con el estudio de Lozano^13^. Teniendo en cuenta que esta especialidad es donde se utiliza con mayor intensidad todo el instrumental rotatorio, es de esperarse este resultado^20^. Sin embargo, se debe considerar que no se revisó el instrumental rotatorio para saber su estado de mantenimiento, marca o modelo utilizado, estas variables podrían influir en el nivel de ruido producido en la práctica clínica.

La especialidad clínica que estuvo en segundo lugar en el nivel de ruido registrado fue odontopediatría; estos resultados del ruido producido, entre otros, se debe al instrumental rotatorio, a lo que se suma el ruido generado por los pacientes pediátricos debido al estrés que les producen los procedimientos durante la atención clínica. Para minimizar estos ruidos se han propuesto diferentes estrategias orientadas a mejorar el comportamiento de los menores de tal manera que se pueda desarrollar de manera apropiada la atención y con ello los niveles de ansiedad y estrés^21^. No obstante, esta situación hace los niveles de ruido producidos en esta especialidad sean más altos que en otras áreas odontológicas^22^. La investigación adelantada por por Voitl sobre la exposición a ruido en la consulta odontopediátrica reportó que la exposición diaria más alta de los pediatras fue de 79 dB con una exposición promedio de 74 dB^23^, datos similares a los reportados en el presente estudio donde el nivel de ruido emitido en el área de pediatría fue de 77 dB.

Por su parte, el estudio de Qsaibati e Ibrahim realizado en la Universidad de Damasco, reportó mediciones de ruido usando como instrumento medidor un micrófono que fue ubicado 15 cm de distancia a una fuente de ruido en áreas preclínicas y clínicas. Los autores informaron que la especialidad de pediatría registró los más altos niveles de ruido (67,37 dB) señalando que los pacientes pediátricos por lo general no cooperan durante la atención clínica^24^.

Según un estudio realizado por Burk y Neitzel de la facultad de odontología de la Universidad de Michigan, se observó que el 4% de las mediciones estandarizadas por los límites máximos de exposición ocupacional de 8 horas superaron los límites permisibles de 85 dB según lo establecido por el Instituto Nacional de Seguridad y Salud Ocupacional, lo cual cuestiona sobre los posibles efectos secundarios del ruido dental en la audición de los estudiantes y personal de odontología y evidencia el riesgo de desarrollar pérdida auditiva inducida por ruido, particularmente en entornos clínicos pediátricos^25^. Los procedimientos clínicos odontológicos realizados en el presente estudio con las especialidades de operatoria dental, rehabilitación oral, endodoncia y odontopediatría se encontraron dentro de los límites permisibles de ruido según el Ministerio de Salud Nacional. No obstante, el día jueves registró los niveles más altos de ruido, debido a que para este día están programadas las prácticas de la especialidad de operatoria dental, la cual muestra los mayores niveles de ruido.

Otros factores evaluados en este estudio fue la jornada y el piso en el que cada especialidad se encontraba llevando a cabo los procesos odontológicos. El análisis estadístico no mostró diferencias significativas con estas variables, lo que indica que la distribución del espacio de las clínicas, así como el horario asignado para cada los procedimientos no influyen en la generación de ruido ambiental.

Los autores destacan que al a fecha este estudio es el primero que se ha realizado en la Universidad Santo Tomás seccional Bucaramanga sede de Floridablanca donde se evaluó el nivel de ruido promedio generado en las clínicas. Adicionalmente, los investigadores contaron con la participación de un profesional del área de ingeniería capacitado en el manejo del sonómetro y quien realizó las mediciones de forma cegada lo que garantizó la objetividad en el registro y reporte de los hallazgos. Como limitaciones del presente estudio los autores reconocen que las mediciones se realizaron entre jueves y sábado ya que es donde se concentra la actividad clínica dejando por fuera los primeros días de la semana y con ello, ausencia de información del ruido los primeros días de la semana; de igual manera, no se evaluó el estado actual y el mantenimiento del material rotatorio así como las marcas respectivas, siendo elementos importantes para establecer posibles asociaciones con el nivel del ruido de acuerdo a lo que ha sido previamente reportado en la literatura.

## Conclusión

Con este trabajo, los autores concluyen que los niveles de ruido generados a partir de los procedimientos clínicos odontológicos de operatoria dental, rehabilitación, endodoncia y odontopediatría se encontraron dentro de los límites permisibles de ruido de acuerdo a los establecido por el Ministerio de Salud de Colombia a la fecha de realización de la investigación. No obstante, dado que el ruido puede acumularse es importante que el personal docente y estudiantes de odontología reconozcan el grado de exposición al que se ven expuestos y, por tanto, adopten medidas de protección auditiva a fin de minimizar el riesgo de pérdida de la audición.
